# Viral Mimetic-Induced Inflammation Abolishes Q-Pathway, but Not S-Pathway, Respiratory Motor Plasticity in Adult Rats

**DOI:** 10.3389/fphys.2019.01039

**Published:** 2019-08-13

**Authors:** Austin D. Hocker, Adrianne G. Huxtable

**Affiliations:** Department of Human Physiology, University of Oregon, Eugene, OR, United States

**Keywords:** respiratory motor plasticity, viral inflammation, polyIC, inflammation, phrenic long-term facilitation

## Abstract

Inflammation arises from diverse stimuli eliciting distinct inflammatory profiles, yet little is known about the effects of different inflammatory stimuli on respiratory motor plasticity. Respiratory motor plasticity is a key feature of the neural control of breathing and commonly studied in the form of phrenic long-term facilitation (pLTF). At least two distinct pathways can evoke pLTF with differential sensitivities to bacterial-induced inflammation. The Q-pathway is abolished by bacterial-induced inflammation, while the S-pathway is inflammation-resistant. Since viral-induced inflammation is common and elicits distinct temporal inflammatory gene profiles compared to bacterial inflammation, we tested the hypothesis that inflammation induced by a viral mimetic (polyinosinic:polycytidylic acid, polyIC) would abolish Q-pathway-evoked pLTF, but not S-pathway-evoked pLTF. Further, we hypothesized Q-pathway impairment would occur later relative to bacterial-induced inflammation. PolyIC (750 μg/kg, i.p.) transiently increased inflammatory genes in the cervical spinal cord (3 h), but did not alter medullary and splenic inflammatory gene expression, suggesting region specific inflammation after polyIC. Dose-response experiments revealed 750 μg/kg polyIC (i.p.) was sufficient to abolish Q-pathway-evoked pLTF at 24 h (17 ± 15% change from baseline, *n* = 5, *p* > 0.05). However, polyIC (750 μg/kg, i.p.) at 3 h was not sufficient to abolish Q-pathway-evoked pLTF (67 ± 21%, *n* = 5, *p* < 0.0001), suggesting a unique temporal impairment of pLTF after viral-mimetic-induced systemic inflammation. A non-steroidal anti-inflammatory (ketoprofen, 12.5 mg/kg, i.p., 3 h) restored Q-pathway-evoked pLTF (64 ± 24%, *n* = 5, *p* < 0.0001), confirming the role of inflammatory signaling in pLTF impairment. On the contrary, S-pathway-evoked pLTF was unaffected by polyIC-induced inflammation (750 μg/kg, i.p., 24 h; 72 ± 25%, *n* = 5, *p* < 0.0001) and was not different from saline controls (65 ± 32%, *n* = 4, *p* = 0.6291). Thus, the inflammatory-impairment of Q-pathway-evoked pLTF is generalizable between distinct inflammatory stimuli, but differs temporally. On the contrary, S-pathway-evoked pLTF is inflammation-resistant. Therefore, in situations where respiratory motor plasticity may be used as a tool to improve motor function, strategies targeting S-pathway-evoked plasticity may facilitate therapeutic outcomes.

## Introduction

Neuroplasticity of the respiratory control system is an important feature of the control of breathing thought to confer stability and adaptation when the respiratory system is challenged (Fuller and Mitchell, 2017). One frequently studied model of adult respiratory neuroplasticity is phrenic long-term facilitation (pLTF), induced by acute intermittent hypoxia (AIH). At least two pathways to pLTF exist: the Q-pathway and the S-pathway (reviewed in Turner et al., 2018). The Q-pathway is serotonin-dependent and induced by moderate AIH (mAIH, 3 × 5 min hypoxic episodes, PaO_2_ 35–45 mmHg) (Baker-Herman and Mitchell, 2002), while the S-pathway is induced by severe AIH (sAIH, PaO_2_ 25–35 mmHg) and involves activation of adenosine receptors (Nichols et al., 2012). The cellular mechanisms of Q- and S-pathway plasticity are distinct (Turner et al., 2018) and the two pathways are differentially affected by adult, systemic inflammation (Agosto-Marlin et al., 2017). Adult Q-pathway plasticity is abolished by low levels of acute, systemic inflammation induced by a TLR4 agonist (lipopolysaccharides, LPS) (Huxtable et al., 2011; Vinit et al., 2011) or 8 h of intermittent hypoxia (Huxtable et al., 2015, 2017) and restored by the non-steroidal anti-inflammatory, ketoprofen (Huxtable et al., 2013, 2015). On the contrary, S-pathway-evoked adult respiratory motor plasticity is inflammation resistant (Agosto-Marlin et al., 2017), and therefore it has the potential to serve as a “backup” pathway to preserve plasticity after inflammation.

Since AIH-induced plasticity is being translated to clinical populations (e.g., after spinal cord injury) (Trumbower et al., 2012, 2017; Hayes et al., 2014) with low-levels of systemic/neuroinflammation, interest in the impact of inflammation on motor plasticity is growing. Studies on inflammation-induced impairments in pLTF have focused on (LPS, bacterial induced-inflammation) (Vinit et al., 2011; Huxtable et al., 2013; Hocker and Huxtable, 2018) or simulated sleep apnea induced by nocturnal intermittent hypoxia (Huxtable et al., 2015, 2017). LPS is the most common model of acute, systemic inflammation, induces a rapid upregulation of brain inflammatory cytokines, and abolishes pLTF within 3 h and for at least 24 h (Huxtable et al., 2013), while nocturnal intermittent hypoxia is a clinically relevant physiological perturbation also abolishing pLTF (Huxtable et al., 2015). However, viral infections are very common (Obasi et al., 2014) and induce unique, but overlapping, inflammatory profiles through TLR3 activation (Reimer et al., 2008). Polyinosinic:polycytidylic acid (polyIC) is an agonist for TLR3 and is used experimentally to investigate the effects of systemic viral inflammation (Nicodemus and Berek, 2010). PolyIC-induced inflammation induces a temporally slower onset of inflammatory genes in the brain compared to LPS (Krasowska-Zoladek et al., 2007; Konat et al., 2009; Yamato et al., 2014), and therefore, it has the potential to have differential effects on pLTF. Since inflammation arises from diverse stimuli throughout our lives, understanding the effects of polyIC on respiratory motor plasticity broadens our understanding of the impact of inflammation on respiratory control. Additionally, while the inflammation sensitivity of Q-pathway-evoked plasticity has been confirmed in two models of systemic inflammation (reviewed in Hocker et al., 2017), the sensitivity of S-pathway-evoked plasticity has only been investigated after LPS-induced inflammation (Agosto-Marlin et al., 2017). Here, we hypothesize polyIC will induce CNS inflammation and abolish Q-pathway, but not S-pathway, evoked pLTF. Further, we hypothesize slower neuroinflammation onset after peripheral polyIC, where Q-pathway-evoked LTF will be abolished at 24 h, but not 3 h.

## Materials and Methods

All experiments were approved by the University of Oregon Institutional Animal Care and Use Committee and conformed to the policies of the National Institute of Health *Guide for the Care and Use of Laboratory Animals*. Male Sprague Dawley Rats (300–400 g; 3–4 months; Envigo, Colony 206) were housed under standard conditions (12:12 h light/dark cycle) with food and water *ad libitum*.

### Drugs and Materials

Polyinosinic:polycytidylic acid (P9582, Sigma Chemical) was dissolved in sterile saline, heated to 50°C, cooled for re-annealing, and injected (intraperitoneal, i.p.) at doses ranging from 250 μg/kg to 2 mg/kg. S-(+) Ketoprofen (Keto, Sigma Chemical) was dissolved in ethanol (50% V/V, 0.39 g/kg) and sterile saline for acute, adult injections (12.5 mg/ml/kg, i.p., 3 h).

### Experimental Groups

To investigate the temporal actions of polyIC and the sufficiency of polyIC to abolish Q-pathway-evoked pLTF, the following groups were used: 3 h polyIC (750 μg/kg, *n* = 5), 24 h polyIC (750 μg/kg, *n* = 4), 24 h polyIC (500 μg/kg, *n* = 4), 24 h polyIC (250 μg/kg, *n* = 4). A time control group consisted of rats from each of the groups: 3 h polyIC (750 μg/kg, *n* = 3), 24 h polyIC (750 μg/kg, *n* = 2), 24 h polyIC (500 μg/kg, *n* = 1), 24 h polyIC (250 μg/kg, *n* = 1).

To investigate if acute, anti-inflammatory treatment restores Q-pathway-evoked pLTF after polyIC, rats were treated ketoprofen (12.5 mg/kg, i.p.) 3 h before electrophysiology experiments. The following experimental groups were used: 24 h vehicle (saline) + Keto (*n* = 4), 24 h polyIC (750 μg/kg) + Keto (*n* = 4), and time controls consisting of two animals from each treatment group (*n* = 4).

To investigate the impact of polyIC-induced inflammation on S-pathway-evoked respiratory pLTF, the following experimental groups were used: 24 h vehicle (*n* = 4), 24 h polyIC (750 μg/kg; *n* = 5), and time controls consisting of two animals from each treatment group (*n* = 4).

### Electrophysiological Studies

Electrophysiological studies have been described in detail previously (Bach and Mitchell, 1996; Baker-Herman and Mitchell, 2002; Hocker and Huxtable, 2018). Briefly, rats were anesthetized with isoflurane, tracheotomized, ventilated (Rat Ventilator, VetEquip^®^), and bilaterally vagotomized. A venous catheter was placed for drug delivery and fluid replacement, and a femoral arterial catheter was used to monitor blood pressure and for arterial blood sampling. Arterial blood samples were assessed (PaO_2_, PaCO_2_, pH, base excess; Siemens RAPIDLAB^®^ 248) during baseline, the first hypoxic response, and 15, 30, and 60 min post-AIH. Rectal temperature was measured (Kent Scientific Corporation) and maintained between 37 and 38°C with a custom heated surgical table. Using a dorsal approach, phrenic nerves were isolated, cut distally, and de-sheathed. Rats were converted to urethane anesthesia (1.8 g/kg i.v.; Sigma-Aldrich), allowed to stabilize for 1 h, and paralyzed with pancuronium bromide (1 mg; Selleck Chemicals). Nerves were bathed in mineral oil and placed on bipolar silver electrodes. Raw nerve recordings were amplified (10 k), filtered (0.1–5 kHz), integrated (50 ms time constant), and recorded (10 kHz sampling rate) for offline analysis (PowerLab and LabChart 8.0, AD Instruments). Apneic and recruitment CO_2_ thresholds were determined by continuous end-tidal CO_2_ monitoring (Kent Scientific Corporation) while changing inspired CO_2_. End-tidal CO_2_ was set 2 mmHg above the recruitment threshold, where baseline PaCO_2_ was established and then maintained within 1.5 mmHg for the duration of the experiment. Blood volume and base excess were maintained (±3 MEq/L) by continuous infusion (0–3 mL/h, i.v.) of hetastarch (0.3%) and sodium bicarbonate (0.99%) in lactated ringers. Experiments were excluded if mean arterial pressure deviated more than 20 mmHg from baseline.

Rats (excluding time control rats) received three, 5-minute bouts of either mAIH (∼10.5% O_2_, PaO_2_ 35–45 mmHg) or sAIH (∼7% O_2_, PaO_2_ 25–35 mmHg). The average frequency and amplitude of 30 consecutive integrated phrenic bursts were assessed during baseline, the first acute hypoxic response, and 15, 30, and 60 min after AIH. Data are presented as the percent relative to baseline and compared using two-way, repeated measures ANOVA with Fisher LSD *post hoc* tests (GraphPad Prism v8). Physiological variables were compared using two-way, repeated measures ANOVA with Tukey’s *post hoc* test. Mean arterial pressure is reported for baseline, the end of the third hypoxic episode, and 60 min after AIH. Acute hypoxic phrenic responses were compared using an ANOVA with Fisher LSD *post hoc* test. Values are presented as mean ± SD.

### RNA Isolation and Quantitative PCR Experiments

Male rats were injected with either vehicle (saline) or polyIC (750 μg/kg, i.p.) 3 or 24 h (*n* = 6 per group) before tissue collection. Rats were anesthetized with isoflurane and perfused with transcardiac PBS (pH 7.4). Ventral cervical spinal cords (C3-C7), medullas, and spleens were dissected and flash frozen until homogenization in TRIzol Reagent (Invitrogen, Carlsbad, CA, United States). RNA was isolated with PureLink columns (Invitrogen, Carlsbad, CA, United States) according to the manufacturer’s protocol. cDNA was reverse transcribed from 1 μg of total RNA using qScript cDNA Synthesis Kit (Quantabio) reverse transcriptase and analyzed using qPCR with PerfeCTa SYBR Green FastMix (Quantabio) on a CFX-384 system (Bio-Rad). Inflammatory gene expression was analyzed in ventral cervical spinal cord, medullary, and spleen homogenates using the following primers:

IL-6: 5′-GTG GCT AAG GAC CAA GAC CA and 5′-GGT TTG CCG AGT AGA CCT CA;

IL-1β: 5′-CTG CAG ATG CAA TGG AAA GA and 5′-TTG CTT CCA AGG CAG ACT TT;

COX-2: 5′-TGT TCC AAC CCA TGT CAA AA and 5′-CGT AGA ATC CAG TCC GGG TA;

TNF-α: 5′-TCC ATG GCC CAG ACC CTC ACA C and 5′-TCC GCT TGG TGG TTT GCT ACG;

iNOS: 5′-AGG GAG TGT TGT TCC AGG TG and 5′-TCT GCA GGA TGT CTT GAA CG;

18s: 5′-CGG GTG CTC TTA GCT GAG TGT CCC G and 5′-CTC GGG CCT GCT TTG AAC AC.

Wherever possible, primers (purchased from Thermo Fisher) were designed to span introns (Primer 3 software). Primer efficiency was assessed by use of standard curves, as previously reported (Crain and Watters, 2015). Expression of inflammatory genes was made relative to 18s ribosomal RNA calculated using the 2^–ΔΔCT^ method (Livak and Schmittgen, 2001).

## Results

### PolyIC Induces Transient Inflammatory Gene Expression in the Ventral Cervical Spinal Cord

**FIGURE 1:**
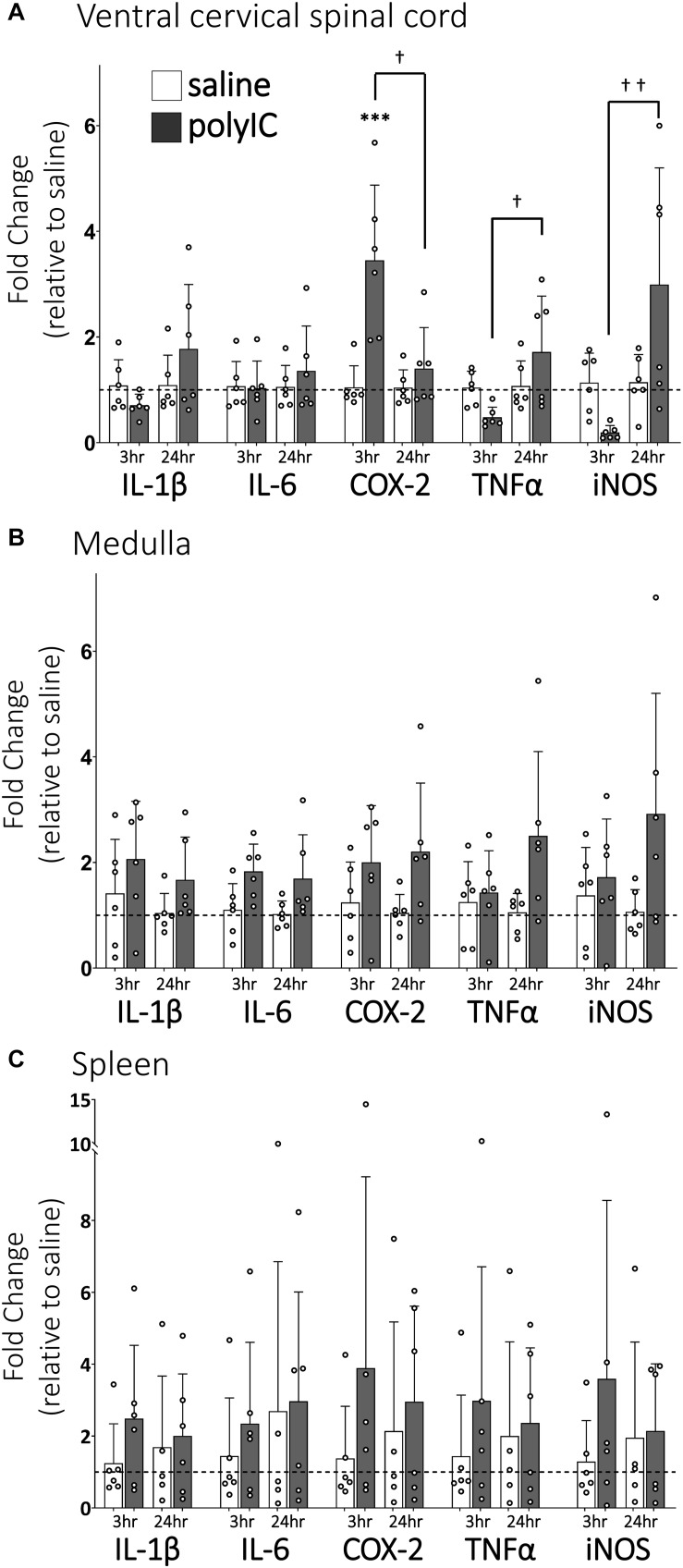
PolyIC (750 μg/kg, i.p.) transiently increases inflammatory gene expression in ventral cervical spinal cord homogenates, but not in the medulla or spleen. Ventral spinal cord homogenate COX-2 mRNA (*n* = 6) significantly increased 3 h after polyIC and returned to baseline by 24 h **(A)**. TNFα and iNOS mRNA significantly increased in the ventral spinal cord at 24 h compared to 3 h after polyIC (*n* = 6 per group), but were not different from saline controls **(A)**. Other inflammatory genes were not significantly altered by polyIC in ventral cervical spinal cords **(A)**, medullas **(B)** or spleens **(C)**. (^∗∗∗^*p* < 0.001 significant difference from saline control, ^†^*p* < 0.01 between groups, ^†⁣†^*p* < 0.001 between groups. ANOVA, Tukey).

Ventral cervical spinal inflammatory gene expression 3 and 24 h after polyIC (750 μg/kg, i.p., Figure 1A) was assessed since previous studies have demonstrated peripheral LPS abolishes Q-pathway-evoked pLTF within 3 h and for at least 24 h (Huxtable et al., 2013), and involves activation of spinal IL-1 receptors (Hocker and Huxtable, 2018). In ventral cervical spinal homogenates, IL-1β, IL-6, TNFα and iNOS gene expression were not significantly different from saline controls at 3 or 24 h (Figure 1A). However, COX-2 gene expression transiently increased at 3 h (*n* = 6, *p* < 0.001, Figure 1A), but returned to baseline by 24 h after polyIC. At 24 h, expression of TNFα and iNOS genes was significantly increased relative to 3-hour polyIC (*p* = 0.01 and *p* = 0.003 respectively), but was not different from 24-hour saline controls (*p* > 0.05). Thus, inflammatory gene expression increases in regions of the central nervous system important for pLTF 3 h after polyIC, with some regions remaining insignificantly elevated at 24 h. Medullary gene expression (Figure 1B) was also assessed to investigate regional differences in CNS inflammatory responses and splenic gene expression (Figure 1C) as a marker of systemic inflammation. In medullary and splenic homogenates, no significant changes in inflammatory genes (IL-6, IL-1β, COX-2, TNF-α, iNOS) were evident at 3 or 24 h (Figures 1B,C), suggesting polyIC induces minimal systemic or medullary inflammation.

### Q-Pathway-Evoked pLTF Is Abolished 24 h After PolyIC

**FIGURE 2:**
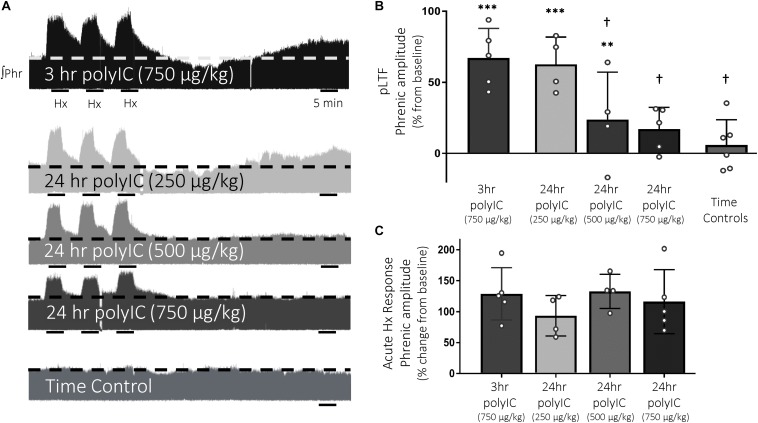
The viral mimetic, polyIC, abolishes Q-pathway-evoked pLTF at 24 h, but not at 3 h. Representative integrated phrenic neurograms after polyIC (250–750 μg/kg, 3 or 24 h, i.p.) **(A)**. Q-pathway-evoked pLTF is evident as the progressive increase in phrenic nerve amplitude from baseline (dashed line) over 60 min following moderate acute intermittent hypoxia (mAIH, 3 × 5 min episodes, PaO_2_ 35–45 mmHg). Group data **(B)** demonstrate 750 μg/kg polyIC was insufficient to abolish Q-pathway-evoked pLTF at 3 h (*n* = 5). However, Q-pathway-evoked pLTF is abolished 24 h after 750 μg/kg polyIC (*n* = 4), but not at lower doses of polyIC (500 μg/kg, *n* = 4; 250 μg/kg, *n* = 4). Time controls (no AIH, *n* = 7) are not different from baseline, but were significantly reduced from 3 h polyIC (750 μg/kg) and 24 h polyIC (250 μg/kg) groups. Acute hypoxic phrenic nerve responses **(C)** were not altered by polyIC at any dose. (^∗∗∗^*p* < 0.0001 significant difference in phrenic amplitude from baseline; ^∗∗^*p* < 0.001 from baseline, ^†^*p* < 0.01 different from 3 h polyIC 750 μg/kg and 24 h polyIC 250 μg/kg, ANOVA RM, Fisher LSD).

Q-pathway-evoked pLTF is evident as the increase in integrated phrenic nerve activity 60 min after mAIH (PaO_2_ 35–45 mmHg) in anesthetized rats (Bach and Mitchell, 1996; Figure 2A). The dose of polyIC (750 μg/kg) sufficient to induce transient cervical spinal inflammatory gene expression was insufficient to abolish Q-pathway-evoked pLTF at 3 h (67 ± 21% change from baseline, *n* = 5, *p* < 0.001, Figure 2B). At 24 h, a dose response to polyIC revealed 750 μg/kg was sufficient to abolish Q-pathway-evoked pLTF (17 ± 15% change from baseline, *n* = 5, *p* = 0.058, Figure 2B). Lower doses of polyIC were insufficient to significantly abolish pLTF (500 μg/kg = 24 ± 33% change from baseline, *n* = 4, *p* = 0.019; 250 μg/kg = 63 ± 19% change from baseline, *n* = 4, *p* < 0.001, Figure 2B). Variability in pLTF magnitude 24 h after 500 μg/kg polyIC suggests this dose is near the threshold to abolish plasticity. Phrenic amplitude in time controls (not receiving mAIH) did not differ from baseline (6 ± 18% change from baseline, *n* = 6, *p* = 0.466, Figure 2B), demonstrating no effect of polyIC on experiment stability. Between groups, phrenic amplitude 60 min after mAIH was significantly reduced (*p* < 0.01) after 24 h polyIC (500 μg/kg) and 24 h polyIC (250 μg/kg, and time controls) compared to 3 h polyIC (750 μg/kg) and 24 h polyIC (250 μg/kg) groups. Thus, polyIC-induced impairment of pLTF occurs slower than LPS-induced impairment and after inflammatory gene expression has returned to baseline. Phrenic nerve responses to moderate hypoxia were not different among groups (Figure 2C).

### Ketoprofen Restores Q-Pathway-Evoked pLTF After PolyIC-Induced Inflammation

**FIGURE 3:**
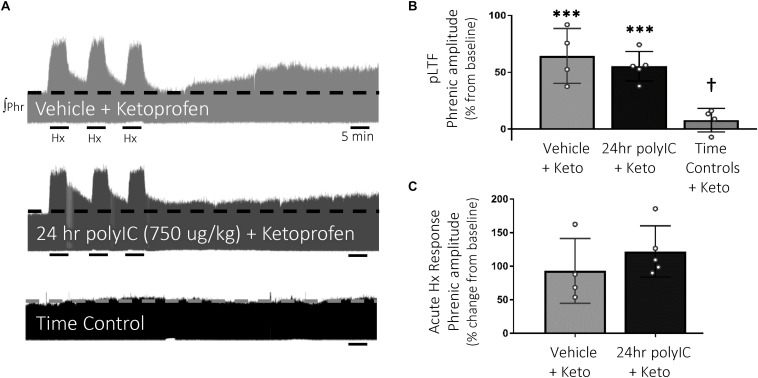
Acute, anti-inflammatory ketoprofen restores Q-pathway-evoked pLTF after polyIC-induced inflammation at 24 h. Representative integrated phrenic neurograms after polyIC (750 μg/kg, i.p., 24 h) and acute, adult ketoprofen (12.5 mg/kg, i.p., 3 h) **(A)**. Q-pathway-evoked pLTF is evident as the progressive increase in phrenic nerve amplitude from baseline (dashed line) over 60 min following moderate acute intermittent hypoxia (mAIH, 3 × 5 min episodes, PaO_2_ 35–45 mmHg). Group data **(B)** demonstrate Q-pathway-evoked pLTF is restored 24 h after 750 μg/kg polyIC by the anti-inflammatory ketoprofen (*n* = 4). Time controls (no mAIH, *n* = 4) are not different from baseline and were significantly reduced from vehicle and 24 h polyIC groups (*n* = 4). Acute hypoxic phrenic nerve responses **(C)** were not altered by polyIC or ketoprofen. (^∗∗∗^*p* < 0.0001 significant difference in phrenic amplitude from baseline; ^†^*p* < 0.001 different from all other groups, ANOVA RM, Fisher LSD).

To test the hypothesis that inflammatory signaling after polyIC (750 μg/kg, 24 h) abolished Q-pathway-evoked pLTF, the anti-inflammatory ketoprofen (12.5 mg/kg, i.p., 3 h) was used to acutely diminish inflammatory signaling (Figure 3A). As expected, Q-pathway-evoked pLTF was evident in vehicle + Ketoprofen treated rats 60 min after mAIH (64 ± 24% change from baseline, *n* = 4, *p* < 0.001, Figure 3B). Ketoprofen restored Q-pathway-evoked pLTF after polyIC (55 ± 13% change from baseline, *n* = 5, *p* < 0.001, Figure 3B), suggesting polyIC-induced inflammation abolishes Q-pathway-evoked pLTF. pLTF was not apparent in time control rats (8 ± 10% change from baseline, *n* = 4, *p* = 0.431, Figure 3B). Phrenic amplitude 60 min after mAIH was significantly greater (*p* < 0.001) in vehicle + Keto and polyIC + Keto groups relative to time controls. Phrenic nerve responses to hypoxia were not different among groups (Figure 3C).

### S-Pathway-Evoked pLTF Is Resistant to PolyIC-Induced Inflammation

**FIGURE 4:**
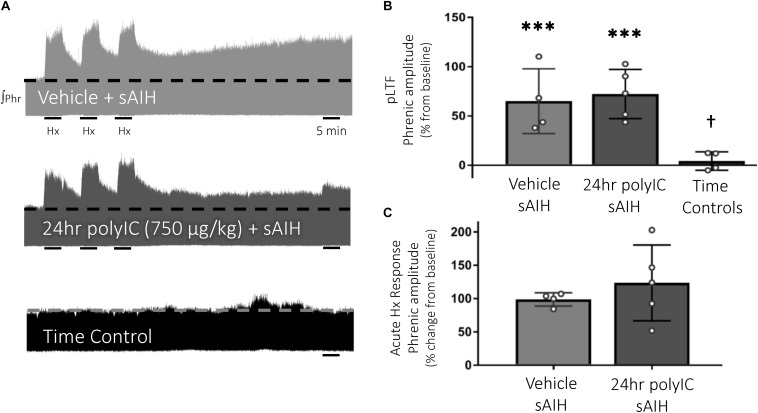
S-pathway-evoked pLTF persists 24 h after the viral mimetic, polyIC. Representative integrated phrenic neurograms after polyIC (750 μg/kg, i.p., 24 h) **(A)**. S-pathway-evoked pLTF is evident as the progressive increase in phrenic nerve amplitude from baseline (dashed line) over 60 min following severe acute intermittent hypoxia (sAIH, 3 × 5 min episodes, PaO_2_ 25–35 mmHg). Group data **(B)** demonstrate S-pathway-evoked pLTF remains 24 h after 750 μg/kg polyIC (*n* = 5). Time controls (no sAIH, *n* = 4) are not different from baseline and were significantly reduced from vehicle (*n* = 4) and 24 h polyIC groups. Acute hypoxic phrenic nerve responses to severe hypoxia **(C)** were not altered by polyIC. (^∗∗∗^*p* < 0.0001 significant difference in phrenic amplitude from baseline; ^†^*p* < 0.001 different from all other groups, ANOVA RM, Fisher LSD).

S-pathway-evoked pLTF was evident as the significant increase in integrated phrenic amplitude 60 min after sAIH (PaO_2_ 25–35 mmHg, Figure 4A) in adult rats treated with vehicle (65 ± 33% change from baseline, *n* = 4, *p* < 0.001, Figure 4B). PolyIC at the same dose undermining Q-pathway-evoked pLTF (750 μg/kg, i.p., 24 h) was insufficient to abolish S-pathway-evoked pLTF (72 ± 25% change from baseline, *n* = 5, *p* < 0.001, Figure 4B), suggesting S-pathway-evoked pLTF is resistant to polyIC-induced inflammation. Phrenic amplitude did not change from baseline in time control rats (4 ± 9% change, *n* = 4, *p* = 0.670, Figure 4B). Both vehicle and polyIC groups had significantly greater phrenic amplitude 60 min after sAIH (*p* = 0.027 and *p* = 0.002, respectively) compared to time controls. Phrenic nerve responses to severe hypoxia were not different among groups (Figure 4C).

Physiological variables for all experimental groups were stable throughout electrophysiology experiments without any unexpected changes (Table 1).

**TABLE 1:** Physiological parameters during electrophysiology experiments.

**Baseline**	**Temperature (°*C*)**	**POa2 (mmHg)**	**PCaO2 (mmHg)**	**pH**	**MAP (mmHg)**
750 μg/kg polyIC (3 h)	37.3 ± 0.2	282 ± 26	44.4 ± 1.1	7.36 ± 0.03	121 ± 14
750 μg/kg polyIC (24 h)	37.5 ± 0.4	268 ± 7	46.7 ± 2.4	7.35 ± 0.03	136 ± 24
500 μg/kg polyIC (24 h)	37.5 ± 0.3	287 ± 25	42.5 ± 3.4	7.38 ± 0.04	125 ± 17
250 μg/kg polyIC (24 h)	37.4 ± 0.3	279 ± 37	45.5 ± 3.9	7.34 ± 0.02	120 ± 26
Time Controls	37.5 ± 0.3	252 ± 29	45.6 ± 1.7	7.37 ± 0.02	123 ± 11
Vehicle + keto	37.5 ± 0.3	261 ± 28	43.8 ± 1.8	7.38 ± 0.02	123 ± 11
polyIC + keto	37.6 ± 0.1	267 ± 43	44.9 ± 2.3	7.35 ± 0.03	120 ± 22
Time Controls + keto	37.4 ± 0.4	246 ± 36	43.2 ± 2.0	7.37 ± 0.01	124 ± 28
Vehicle + sAIH	37.4 ± 0.3	262 ± 18	44.6 ± 2.4	7.39 ± 0.02	117 ± 5
polyIC + sAIH	37.3 ± 0.2	255 ± 29	44.9 ± 2.4	7.39 ± 0.03	118 ± 18
Time Controls	37.8 ± 0.1	260 ± 11	41.8 ± 1.1	7.4.0 ± 0.03	94 ± 19
**Hypoxia**					
750 μg/kg polyIC (3 h)	37.5 ± 0.2	42 ± 3^ab^	43.6 ± 1.3	7.35 ± 0.02	98 ± 23^a^
750 μg/kg polyIC (24 h)	37.4 ± 0.4	41 ± 4^ab^	46.3 ± 1.8	7.34 ± 0.03	98 ± 35^ab^
500 μg/kg polyIC (24 h)	37.3 ± 0.2	39 ± 2^ab^	41.4 ± 4.3	7.38 ± 0.03	90 ± 30^a^
250 μg/kg polyIC (24 h)	37.5 ± 0.4	41 ± 4^ab^	45.5 ± 4.1	7.33 ± 0.02	102 ± 27
Time Controls	37.5 ± 0.3	260 ± 31^c^	44.5 ± 1.9	7.37 ± 0.02	78 ± 29
Vehicle + keto	37.4 ± 0.4	35 ± 6^ab^	42.4 ± 1.6	7.36 ± 0.01	78 ± 29^*ab*^
polyIC + keto	37.7 ± 0.2	41 ± 4^ab^	44.1 ± 1.8	7.33 ± 0.02	80 ± 30^b^
Time Controls + keto	37.4 ± 0.4	260 ± 14^c^	43.5 ± 2.0	7.38 ± 0.01	124 ± 28
Vehicle + sAIH	37.4 ± 0.3	29 ± 2^ab^	43.7 ± 3.0	7.37 ± 0.04	70 ± 14^ab^
polyIC + sAIH	37.4 ± 0.2	31 ± 4^ab^	43.8 ± 1.2	7.37 ± 0.04	85 ± 15^b^
Time Controls	37.8 ± 0.2	252 ± 8^c^	41.0 ± 1.3	7.41 ± 0.01	94 ± 20
**60 min**					
750 μg/kg polyIC (3 h)	37.4 ± 0.4	272 ± 10	44.3 ± 1.6	7.37 ± 0.01	130 ± 12
750 μg/kg polyIC (24 h)	37.6 ± 0.2	238 ± 43	47.2 ± 2.8	7.34 ± 0.03	136 ± 32
500 μg/kg polyIC (24 h)	37.8 ± 0.3	250 ± 35	42.7 ± 3.5	7.38 ± 0.03	126 ± 20
250 μg/kg polyIC (24 h)	37.6 ± 0.3	247 ± 37	45.6 ± 3.9	7.33 ± 0.04	122 ± 21
Time Controls	37.6 ± 0.2	266 ± 66	45.2 ± 2.3	7.39 ± 0.02	114 ± 7
Vehicle + keto	37.5 ± 0.5	242 ± 40	44.9 ± 1.8	7.38 ± 0.02	114 ± 7
polyIC + keto	37.4 ± 0.3	268 ± 18	45.2 ± 2.3	7.34 ± 0.02	109 ± 21
Time Controls + keto	37.8 ± 0.0	236 ± 29	42.8 ± 1.8	7.39 ± 0.02	116 ± 28
Vehicle + sAIH	37.7 ± 0.4	250 ± 17	44.3 ± 2.0	7.38 ± 0.02	112 ± 13
polyIC + sAIH	37.6 ± 0.3	240 ± 19	44.9 ± 1.7	7.38 ± 0.03	115 ± 21
Time Controls	37.5 ± 0.3	252 ± 19	42.0 ± 1.0	7.40 ± 0.02	93 ± 20

*MAP, mean arterial pressure; P_*a*_O_2_, arterial oxygen pressure; P_*a*_CO_2_, arterial carbon dioxide pressure. Statistical comparisons: ANOVA RM, Tukey’s *post hoc*: ^*a*^different from 60 min timepoint within group, ^*b*^different from baseline within group, ^*c*^different from all other groups within timepoint.*

## Discussion

To further our understanding of inflammation-induced impairment in adult respiratory motor plasticity, we investigated the impact of viral-mimetic-induced inflammation on Q- and S-pathway-evoked pLTF. Here, we demonstrate inflammatory impairment in respiratory motor plasticity is generalizable to viral-mimetic-induced inflammation. The viral-mimetic, polyIC, induced a transient increase in cervical spinal inflammatory gene expression and impaired Q-pathway-evoked pLTF. However, the time course of inflammatory impairment of pLTF is slower after polyIC compared to bacterial-induced inflammation (Huxtable et al., 2013). Additionally, S-pathway-evoked pLTF is resistant to polyIC-induced systemic inflammation, suggesting preferentially targeting S-pathway-evoked motor plasticity is an effective strategy to elicit a therapeutic benefit in conditions with coincident inflammation.

Polyinosinic:polycytidylic acid is a commonly used viral-mimetic binding to TLR3 (Nicodemus and Berek, 2010) and is used here to induce systemic inflammation. In this study, 750 μg/kg polyIC (i.p.) was sufficient to induce ventral cervical spinal homogenate inflammatory gene expression within 3 h, resolving by 24 h. However, splenic inflammatory gene expression (a marker of systemic inflammation) showed no significant increase in inflammatory genes 3 or 24 h after polyIC, suggesting this dose of polyIC induces relatively low-grade inflammation and has region-specific effects. These regional differences could be a result of differing mechanisms of peripheral to central inflammatory transmission, such as vagal transmission (Laye et al., 1995; Balan et al., 2011) or transmission through the blood brain barrier (Siljehav et al., 2012). Additionally, the blood-spinal cord barrier is more permeable to peripheral cytokines than the blood brain barrier (Pan et al., 1997), suggesting increased spinal inflammation could be a result of faster peripheral inflammatory signal transmission from the periphery to the spinal cord relative to the medulla. However, our understanding of the detailed mechanisms underlying peripheral polyIC-induced central inflammation remain unclear (Dantzer, 2009; Konat et al., 2009) and warrant further investigation.

The time course of inflammatory gene expression after peripheral polyIC presented here is similar to increased cervical spinal inflammatory gene expression observed after peripheral LPS, which is elevated at 3 h and similarly resolved by 24 h (Huxtable et al., 2013). However, the magnitude of gene upregulation in spinal homogenates was substantially less following polyIC compared to LPS (Huxtable et al., 2013), suggesting 750 μg/kg polyIC is likely inducing a lower level of CNS inflammation. Yet, an inherent difficulty in comparing distinct inflammatory stimuli is identifying comparable doses. PolyIC dose-response experiments in rats demonstrate 750 μg/kg (i.p.) is sufficient to upregulate circulating cytokines and induce fever through an IL-1 dependent mechanism (Fortier et al., 2004). Therefore, 750 μg/kg (i.p.) polyIC is comparable to 100 μg/kg LPS since both induce similar febrile responses in rats (Boisse et al., 2005), and induce spinal inflammatory gene expression within 3 h, but only LPS is sufficient to impair pLTF at 3 h.

While LPS and polyIC both induce MAPKs, NF-κB, and increase pro-inflammatory gene expression, peripheral polyIC induces a slower onset and longer lasting upregulation of inflammatory gene expression in the CNS compared to LPS (Krasowska-Zoladek et al., 2007; Konat et al., 2009; Yamato et al., 2014). These differences may arise from differential downstream signaling mechanisms of polyIC compared to LPS, including different TLR adaptor signaling (Matsumoto and Seya, 2008; Zhu et al., 2016) and TLR3-independent targets of polyIC (Matsumoto and Seya, 2008; McCartney et al., 2009; Starkhammar et al., 2012; Hu et al., 2013; Zhu et al., 2016). Alternatively, a temporal delay in polyIC-induced inflammatory protein expression may result in delayed or insufficient inflammation to impair plasticity 3 h after polyIC. While it is common for inflammatory gene expression to correlate with inflammatory protein expression, other post-translational regulatory mechanisms influence the strength of this correlation (Guo et al., 2008; Du et al., 2014). However, comparisons of polyIC and LPS induced inflammatory changes in both mRNA and protein suggest the temporal responses of both are delayed after polyIC relative to LPS (Reimer et al., 2008). Additionally, we cannot rule out inflammatory changes in isolated cell types being diluted in homogenate samples, such as the changes seen in isolated microglia after LPS (Huxtable et al., 2013), nor the possibility of unmeasured inflammatory molecules playing an important role(s) in undermining plasticity. Any or all of these differences likely contribute to different time scales of pLTF impairment after polyIC (24 h) compared to LPS (3 and 24 h).

Q-pathway-evoked pLTF was not impaired 3 h after polyIC, yet COX-2 gene expression transiently increased at 3 h. Despite inducing neuroinflammation in regions of the CNS relevant to respiratory plasticity (Baker-Herman et al., 2004), it was not sufficient to impair plasticity. These findings support previous work demonstrating the inflammatory impairment of Q-pathway-evoked pLTF is COX-independent (Huxtable et al., 2017). However, 750 μg/kg polyIC was sufficient to abolish Q-pathway-evoked pLTF at 24 h, when all measured inflammatory genes returned to baseline. Such findings match the results of Fortier et al. (2004), demonstrating 750 μg/kg polyIC was just above the threshold required to induce brain-mediated febrile responses. Thus, 750 μg/kg polyIC is above the threshold dose inducing CNS inflammatory responses and abolishing Q-pathway respiratory motor plasticity. Overall, these findings are similar to those observed 24 h after LPS-induced inflammation (Huxtable et al., 2013), demonstrating the inflammatory impairment in Q-pathway-evoked plasticity is generalizable between inflammatory stimuli.

**FIGURE 5:**
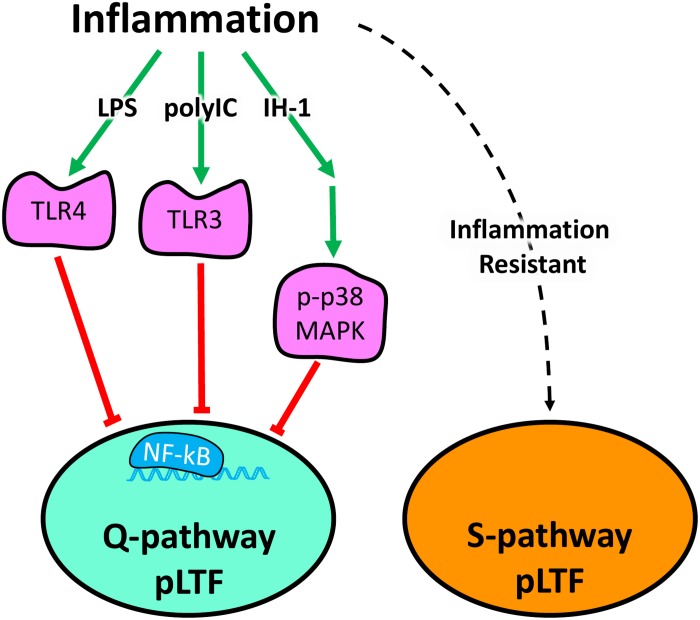
Schematic summarizing diverse inflammatory stimuli impair Q-pathway, but not S-pathway, adult respiratory motor plasticity. Lipopolysaccharides (LPS), polyinosinic:polycytidylic acid (polyIC), and one night of intermittent hypoxia (IH-1) activate peripheral inflammatory pathways and abolish Q-pathway-evoked pLTF. LPS and polyIC activate TLR4 and TLR3, respectively, to induce NF-kB activation and impair Q-pathway-evoked pLTF. IH-1-induced inflammation activates p38 MAPK and NF-kB to impair Q-pathway-evoked respiratory motor plasticity. Thus, multiple, diverse low-level inflammatory stimuli impair Q-pathway-evoked pLTF. S-pathway-evoked pLTF, however, is resistant to both LPS and polyIC-induced inflammation, suggesting it may act as a “backup” pathway after inflammation.

Acute ketoprofen after polyIC-induced inflammation restored Q-pathway-evoked pLTF, confirming the impairment of pLTF is inflammation-dependent. Ketoprofen inhibits both COX and NF-κB (Cashman, 1996; Yin et al., 1998). However, as COX inhibition does not restore Q-pathway-evoked pLTF after inflammation (Huxtable et al., 2017), the restoration of pLTF from ketoprofen is likely due to NF-κB inhibition, though we cannot rule out other off-target effects of ketoprofen. Regardless, our results indicate the viral mimetic polyIC induces cervical spinal inflammation and impairs Q-pathway-evoked pLTF in an inflammation-dependent manner. Previous work demonstrated the acute impairment of pLTF is downstream of serotonin receptors, upstream of BDNF signaling (Agosto-Marlin et al., 2018), and involves IL-1 receptors (Hocker and Huxtable, 2018) and p-38 MAPK activation (Huxtable et al., 2015). Future studies should further investigate more detailed cellular mechanisms by which inflammatory signaling impairs Q-pathway-evoked respiratory motor plasticity after both LPS- and polyIC-induced inflammation (summarized in Figure 5).

S-pathway-evoked pLTF is a distinct molecular signaling pathway inducing facilitation of phrenic motor output and elicited by severe AIH through adenosine 2A receptor activation (Nichols et al., 2012). While Q-pathway-evoked pLTF is abolished by low-levels of inflammation from diverse stimuli (Huxtable et al., 2013, 2015), S-pathway-evoked pLTF is resistant to LPS-induced inflammation (Agosto-Marlin et al., 2017). Here, we demonstrate the inflammation resistance of S-pathway-evoked pLTF after polyIC-induced inflammation (Figure 5). These findings contribute to the possibility S-pathway-evoked pLTF can serve as a “backup” mechanism to motor plasticity during low-level inflammation, even after Q-pathway-evoked pLTF is impaired.

In summary, polyIC impairs Q-pathway, but not S-pathway-evoked, respiratory motor plasticity. Further, the inflammatory impairment of Q-pathway plasticity after polyIC relative to LPS is temporally delayed, though we do not have a detailed regional, time course of the inflammatory response following either polyIC or LPS. Such time course studies would facilitate a mechanistic understanding of the inflammatory impairment of Q-pathway plasticity. While the present studies were all performed in urethane-anesthetized rats, cellular mechanisms evoking respiratory plasticity appear similar between pLTF and ventilatory LTF in freely behaving animals (McGuire et al., 2004, 2005, 2008), suggesting findings may be generalizable to ventilatory LTF. The present study focused on the impact of polyIC in adult male rats, highlighting the need for additional studies in females. However, the lack of sex differences observed in adults after neonatal inflammation (Hocker et al., 2019), suggest females would respond similarly to polyIC-induced inflammation. It is important to note pLTF in females is altered by cycling estrogen levels (Dougherty et al., 2017), which may also influence CNS inflammatory responses (Arakawa et al., 2014). Thus, future studies should address potential sex differences in the acute inflammatory impairment of pLTF and the inflammatory resistance of S-pathway-evoked plasticity in females.

## Conclusion

This is the first study to evaluate the impact of viral-mimetic-induced inflammation on adult respiratory motor plasticity and demonstrates respiratory plasticity is profoundly sensitive to diverse inflammatory stimuli. Our results suggest the sensitivity of Q-pathway-evoked pLTF to inflammation is not stimulus specific and is impaired by viral, bacterial, and physiological inflammatory stimuli (Figure 5). Further, S-pathway-evoked pLTF is resistant to viral mimetic-induced inflammation, supporting the notion S-pathway-evoked plasticity could compensate for Q-pathway impaired plasticity after inflammation (Figure 5). Therefore, inflammation, which is common during disease and injury, can abolish the therapeutic potential of respiratory motor plasticity (Trumbower et al., 2012, 2017; Hayes et al., 2014; Mateika and Komnenov, 2017) and leave patients vulnerable to respiratory failure. Stimuli which preferentially elicit S-pathway-evoked plasticity are more likely to provide therapeutic benefit after inflammation.

## Data Availability

The raw data supporting the conclusion of this manuscript will be made available by the authors, without undue reservation, to any qualified researcher.

## Ethics Statement

This study was performed in strict accordance with the recommendations in the Guide for the Care and Use of Laboratory Animals of the National Institutes of Health. All of the animals were handled according to approved institutional animal care and use committee protocols (#18-02) of the University of Oregon. All surgeries were performed under isoflurane or urethane anesthesia and every effort was made to minimize pain, distress, or discomfort.

## Author Contributions

Both authors contributed conception and design of the study, and revised, read and approved the submitted manuscript version. AH collected the data and performed the statistical analysis, and wrote the first draft of the manuscript.

## Conflict of Interest Statement

The authors declare that the research was conducted in the absence of any commercial or financial relationships that could be construed as a potential conflict of interest.
